# Vorasidenib bei niedriggradigen Gliomen – neues Therapieprinzip mit offenen Fragen zu den Langzeitergebnissen

**DOI:** 10.1007/s00066-023-02179-2

**Published:** 2023-11-20

**Authors:** Clemens Seidel, Nils H. Nicolay

**Affiliations:** Klinik und Poliklinik für Strahlentherapie, Universitätsklinikum Leipzig, Stephanstraße 9a, 04103 Leipzig, Deutschland

## Hintergrund

Isocitratdehydrogenase(IDH)-mutierte niedriggradige Gliome (WHO-Grad 2) sind maligne Hirntumoren, die beträchtliche Einschränkungen und vorzeitige Todesfälle verursachen. Vorasidenib, ein oral verfügbarer hirngängiger Inhibitor der mutierten Enzyme IDH1 und IDH2, zeigte in Voruntersuchungen Aktivität bei IDH-mutierten Gliomen.

## Methoden

Im Rahmen einer doppelblinden Phase-3-Studie wurden Patienten mit residuellem oder rezidiviertem IDH-mutiertem WHO-Grad-2-Gliom mit alleiniger operativer Vortherapie im randomisierten Setting entweder mit oralem Vorasidenib (40 mg einmal täglich) oder gematchtem Placebo in 28-tägigen Zyklen behandelt. Primärer Endpunkt war das auf MRT-Bildgebung basierende progressionsfreie Überleben (PFS) nach verblindeter zentraler Einschätzung durch ein unabhängiges Review-Komitee. Ein weiterer wesentlicher sekundärer Endpunkt war die Zeit bis zur nächsten tumorspezifischen Therapie. Ein Crossover zu Vorasidenib von Placebo war bei bildgebend bestätigter Erkrankungsprogression erlaubt. Sicherheitsparameter wurden erfasst.

## Ergebnisse

Insgesamt 331 Patienten wurden zwischen einer Einnahme von Vorasidenib (168 Patienten) und Placebo (163 Patienten) randomisiert. Nach medianem Follow-up von 14,2 Monaten erhielten noch 226 Patienten (68,3 %) Vorasidenib oder Placebo. Das PFS war in der Vorasidenibgruppe im Vergleich zur Placebogruppe signifikant verlängert (medianes PFS 27,7 Monate vs. 11,1 Monate); die Hazard Ratio für Erkrankungsprogression oder Tod betrug 0,39 (95 %-Konfidenzintervall 0,27–0,56, *p* < 0,001). Die Zeit bis zur nächsten therapeutischen Intervention war in der Vorasidenibgruppe verglichen mit der Placebogruppe ebenfalls bei einer Hazard Ratio von 0,26 signifikant verlängert (95 %-Konfidenzintervall 0,15–0,43, *p* < 0,001). Schwerwiegende unerwünschte Ereignisse traten bei 22,8 % der Vorasidenibpatienten und bei 13,5 % der mit Placebo behandelten Patienten auf. Bei 9,6 % der mit Vorasidenib behandelten Patienten waren Erhöhungen der Alanin-Aminotransferase ≥ Grad 3 zu beobachten (Placebopatienten: 0 %).

## Schlussfolgerung der Autoren

Bei Patienten mit IDH-mutierten WHO-Grad-2-Gliomen verbesserte Vorasidenib signifikant das progressionsfreie Überleben und verzögerte die Zeit bis zur nächsten Intervention.

## Kommentar

Isocitratdehydrogenase(IDH)-Antagonisten stellen eine neue Substanzklasse dar, die sich bei akuter myeloischer Leukämie, Cholangiokarzinomen und IDH-mutierten, niedriggradigen Gliomen in der klinischen Prüfung und klinischen Anwendung befindet. Durch die in der Genese von niedriggradigen Gliomen früh stattfindende treibende IDH1/2-Mutation wird das Enzym IDH1/2 enthemmt und intrazellulär statt α‑Ketoglutarat exzessiv 2‑Hydroxyglutarat gebildet, welches als Onkometabolit zu epigenetischer Dysregulation (G-CIMP-Methylierungs-Phänotyp) und einer spezifischen Biologie in IDH1/2-mutierten Gliomen führt [1]. In klinischen Vorstudien wurde gezeigt, dass der Einsatz des sehr hirngängigen Vorasidenib die Konzentration von Hydroxyglutarat in IDH-mutierten Tumorzellen deutlich reduzieren kann und bei Tumoren ohne Kontrastmittelanreicherung im Gegensatz zu aggressiveren kontrastmittelanreichernden Tumoren progressionsfreie Intervalle nach Einnahme von Vorasidenib möglich sind [2, 3]. Insofern stehen die nun vorgelegten ersten Ergebnisse der Phase-3-INDIGO-Studie auf einem soliden translationalen Fundament.

In die vorliegende Studie [4] wurden Patienten > 12 Jahre mit niedriggradigen IDH1/2-mutierten Astrozytomen bzw. Oligodendrogliomen und niedrigem Risikoprofil eingeschlossen, die nach momentaner Praxis Kandidaten für eine Watch-and-wait-Strategie oder Radiotherapie sind. Hervorzuhebende Einschlusskriterien waren eine zentral nachgewiesene IDH1/2-Mutation, ein residueller Tumor oder ein Tumorrezidiv mit messbarer Ausdehnung in T2-Sequenzen von minimal 1 cm in zwei Achsen und eine Tumorresektion als einzige Vortherapie innerhalb der letzten 5 Jahre. Die Kontrastmittelaufnahme im Tumor musste minimal und non-nodulär imponieren und nach RANO-Kriterien nicht messbar sein. Klinische Faktoren (wie z. B. unkontrollierte epileptische Anfälle), die nicht zum Niedrigrisikoprofil passen, stellten ein Ausschlusskriterium dar.

Die Studie war als doppelblinde, placebokontrollierte, randomisierte internationale Studie mit zentralem, verblindetem bildgebendem Review nach RANO-LGG konzipiert. Nach Tumorprogression erfolgte in der Placebogruppe meist ein Crossover zu Vorasidenib. Die Studie wurde nach Empfehlung des Data- und Safety-Komitees aufgrund von „early efficacy“ für den primären Endpunkt (PFS) und den zentralen sekundären Endpunkt (Zeit bis zur nächsten Intervention) nach der zweiten präspezifizierten Interimsanalyse nach 14 Monaten entblindet, allen Placebopatienten wurde ein Crossover angeboten. Ergebnisse dieser Interimsanalyse sind Gegenstand des publizierten Manuskripts. Die Endpunkte PFS (für Progression oder Tod) und Zeit bis zur nächsten Tumortherapie wurden mit einer Hazard Ratio (HR) für das PFS von 0,39 (*p* < 0,001, stratifizierter „log-rank“) und für die Zeit bis zur nächsten Intervention (HR 0,26, *p* < 0,001, stratifizierter „log-rank“) deutlich erfüllt: Kalkuliert wurde beim Design der Studie für das PFS mit einer HR von 0,6, *p* < 0,025. Das mediane Patientenalter der eingeschlossenen Patienten lag bei 40 Jahren (Minimum: 16 Jahre), der Karnofsky-Index betrug bei mehr als der Hälfte der Patienten 100 %. Die Verteilung zwischen Astrozytomen und Oligodendrogliomen war in etwa ausgeglichen. Im Median lag bei Einschluss in die Studie die Erstdiagnose der Erkrankung in beiden Armen 2,5 bzw. 2,9 Jahre zurück, die Mehrheit der Patienten hatte Tumoren mit einer initialen maximalen Ausdehnung > 2 cm. Bei 25 % der Patienten wurde vor Studieneinschluss mehr als eine Tumorresektion durchgeführt. Die klinischen Variablen waren balanciert zwischen beiden Therapiegruppen. Eine Überlegenheit von Vorasidenib war in allen Subgruppen nachweisbar, insbesondere auch unabhängig vom 1p19q-Deletionsstatus, d. h., Patienten mit Oligodendrogliomen und Astrozytomen profitierten gleichermaßen von der Studientherapie.

Hauptnebenwirkungen der als Dauertherapie ausgelegten Vorasidenibbehandlung waren relevante Erhöhungen der Leberwerte (insbesondere der ALAT), die bei immerhin 10 % der Patienten auftraten. Bei etwa 14 % der Patienten waren Dosisreduktionen oder Abbrüche im Therapiearm erforderlich. Lebensqualitätsdaten sind noch nicht veröffentlicht.

Relevante Limitationen der Vorasidenibstudientherapie ergeben sich unter anderem daraus, dass nur bei ca. 11 % der Patienten Verkleinerungen der Tumoren zu beobachten waren (Darstellung nur im Supplement der Studie), also eine Größenabnahme der Tumoren eher nicht zu erwarten ist. Hier sind nach Strahlentherapie oder alkylierender Chemotherapie deutlich höhere Remissionsraten beschrieben [5, 6]. Auch die Remissionsdauer ist beim Einsatz einer Strahlentherapie oder von Temozolomid als Monotherapie mit ca. 40 Monaten deutlich länger [7]. Die nach der Studie von Buckner et al. bei Grad-2-Gliomen als Standard angesehene Kombinationstherapie aus Bestrahlung und PCV-Chemotherapie erreichte ein medianes progressionsfreies Überleben von 10,4 Jahren (medianes Gesamtüberleben 13,3 Jahre; [8]). Insofern sind die mit Vorasidenib erreichten Zeiten der Erkrankungsstabilisierung als sehr kurz zu betrachten. Ein deutlicher Mehrwert der Vorasidenibtherapie läge vor, wenn sich durch einen frühen Einsatz nicht nur Progressionen verschieben, sondern auch sekundäre Malignisierungen langfristig vermeiden lassen könnten. Hierzu wird die Studie aufgrund der Übernahme von Patienten in den Vorasidenibarm (Crossover) allerdings kaum vergleichende Betrachtungen erlauben. Der aktuell veröffentlichte mediane Nachbeobachtungszeitraum von 14 Monaten ist durch die gewählten Studienendpunkte sicherlich gerechtfertigt, aber für die Erkrankungsdauer von LGG eine äußerst kurze Zeit. Zur Vorasidenibtherapie ist zusätzlich einschränkend zu sagen, dass über die Wirksamkeit einer Radiochemotherapie bei LGG nach Vorasidenibeinsatz keine Daten existieren und auch hierzu die Veröffentlichung von weiteren Langzeitdaten der Studie unbedingt erforderlich ist.

Bei gegenwärtigen und zukünftigen Diskussionen zur Therapiewahl bei niedriggradigen Gliomen muss die gegenwärtige Evidenz zur Langzeitneurotoxizität immer mit bedacht werden. Bei der größten hierzu prospektiv untersuchten Kohorte aus 195 Patienten mit LGG wurde 6 Jahre nach normofraktionierter Bestrahlung kein wesentliches neurokognitives Defizit festgestellt, 12 Jahre nach Bestrahlung war allerdings eine Beeinträchtigung kognitiver Domänen im Vergleich zu nichtbestrahlten Patienten nachweisbar [9, 10]. In einer aktuell veröffentlichten Untersuchung des deutschen Gliom-Netzwerks, für die 75 Patienten mit malignen Gliomen ebenfalls prospektiv longitudinal untersucht wurden, war bei 27 Patienten mit vorhandenen Bestrahlungsplänen im Median 7 Jahre nach Bestrahlung bei einer mittleren Hippocampusdosis < 10 Gy eine stabile oder verbesserte Leistung in allen kognitiven Domänen festzustellen, während bei Patienten mit mittleren Hippocampusdosen > 50 Gy (d. h. bei Bestrahlungen in direkter Nähe zum Hippocampus) eine Tendenz zur Verschlechterung in 4/8 Domänen festzustellen war [11]. Die Autoren folgerten, dass eine multimodale Gliomtherapie die Neurokognition weniger beeinträchtigt als bisher angenommen. Insgesamt erscheint also unter Berücksichtigung der Studienlage zur Neurokognition eine sorgsam nach Stand der Technik durchgeführte kombinierte Radiochemotherapie bei LGG als wirksam und ausreichend sicher und sollte außerhalb von Studien nicht unkontrolliert unterlassen werden. Systematische Daten zur Langzeittoxizität von Vorasidenib stehen noch aus.

### Fazit

Die medikamentöse IDH-Inhibition ist ein interessantes neues Therapieprinzip für niedriggradige IDH1/2-mutierte Gliome mit niedrigem Risikoprofil, die bisher nur engmaschigen Kontrollen unterzogen werden. Wie substanziell ein Vorteil durch die Einnahme von Vorasidenib im Verlauf dieser oft über viele Jahre verlaufenden Erkrankungen – auch für andere Risikogruppen – sein wird, ist allerdings noch abzuwarten. Bis auf Weiteres bleibt die postoperative Bestrahlung mit konsekutiver PCV-Chemotherapie als Therapiestandard bestehen.

*Clemens Seidel und Nils H. Nicolay, Leipzig*

## References

[1] Turcan S, Rohle D, Goenka A (2012). IDH1 mutation is sufficient to establish the glioma hypermethylator phenotype. Nature.

[2] Mellinghoff IK, Lu M, Wen PY (2023). Vorasidenib and ivosidenib in IDH1-mutant low-grade glioma: a randomized, perioperative phase 1 trial. Nat Med.

[3] Mellinghoff IK, Penas-Prado M, Peters KB (2021). Vorasidenib, a dual inhibitor of mutant IDH1/2, in recurrent or progressive glioma; results of a first-in-human phase I trial. Clin Cancer Res.

[4] Mellinghoff IK, van den Bent MJ, Blumenthal DT (2023). Vorasidenib in IDH1- or IDH2-mutant low-grade glioma. N Engl J Med.

[5] Bauman G, Pahapill P, Macdonald D (1999). Low grade glioma: a measuring radiographic response to radiotherapy. The Canadian journal of neurological sciences. J Can Sci Neurol.

[6] Rudà R, Pellerino A, Pace A (2019). Efficacy of initial temozolomide for high-risk low grade gliomas in a phase II AINO (Italian Association for Neuro-Oncology) study: a post-hoc analysis within molecular subgroups of WHO 2016. J Neurooncol.

[7] Baumert BG, Hegi ME, van den Bent MJ (2016). Temozolomide chemotherapy versus radiotherapy in high-risk low-grade glioma (EORTC 22033-26033): a randomised, open-label, phase 3 intergroup study. Lancet.

[8] Buckner JC, Shaw EG, Pugh SL (2016). Radiation plus procarbazine, CCNU, and vincristine in low-grade glioma. N Engl J Med.

[9] Klein M, Heimans JJ, Aaronson NK (2002). Effect of radiotherapy and other treatment-related factors on mid-term to long-term cognitive sequelae in low-grade gliomas: a comparative study. Lancet.

[10] Douw L, Klein M, Fagel SS (2009). Cognitive and radiological effects of radiotherapy in patients with low-grade glioma: long-term follow-up. Lancet.

[11] Pertz M, Schlömer S, Seidel C (2023). Long-term neurocognitive function and quality of life after multimodal therapy in adult glioma patients: a prospective long-term follow-up. Oncology.

